# Sheep and Cattle Reservoirs in the Highest Human Fascioliasis Hyperendemic Area: Experimental Transmission Capacity, Field Epidemiology, and Control Within a One Health Initiative in Bolivia

**DOI:** 10.3389/fvets.2020.583204

**Published:** 2020-10-27

**Authors:** Santiago Mas-Coma, Paola Buchon, Ilra R. Funatsu, Rene Angles, Patricio Artigas, M. Adela Valero, M. Dolores Bargues

**Affiliations:** ^1^Departamento de Parasitología, Facultad de Farmacia, Universidad de Valencia, Valencia, Spain; ^2^Unidad de Limnología, Instituto de Ecología, Universidad Mayor de San Andrés, La Paz, Bolivia; ^3^Cátedra de Parasitología, Facultad de Medicina, Universidad Mayor de San Andrés, La Paz, Bolivia

**Keywords:** *Fasciola hepatica*, sheep and cattle, reservoirs, transmission, epidemiology, human hyperendemic, very high altitude, Bolivia

## Abstract

The Northern Bolivian Altiplano is the human fascioliasis hyperendemic area where the highest prevalences and intensities of infection by *Fasciola hepatica* in humans have been reported. Four animal species are the reservoir species for *F. hepatica* in this area, namely, sheep, cattle, pigs, and donkeys. Livestock for the Aymara inhabitants is crucial because vegetable cultures are not viable due to the inhospitality of the very high altitude of 3,820–4,100 m. A One Health initiative has been implemented in this area in recent years, as the first such control action in a human endemic area ever. Among the different control axes included, special focus is devoted to the two main reservoirs sheep and cattle. Egg embryonation, miracidial infectivity, intramolluscan development, cercarial production, infected snail survival, and metacercarial infectivity were experimentally studied in altiplanic sheep and cattle isolates. These laboratory studies were performed using altiplanic isolates of the lymnaeid species *Galba truncatula*, the only vector present in the hyperendemic area. Experiments were made at constant 12 h day/12 h night and varying 20/20°C and 22/5°C photoperiods. Infections were implemented using mono-, bi-, and trimiracidial doses. Results demonstrate that sheep and cattle have the capacity to assure *F. hepatica* transmission in this very high-altitude area. Field surveys included prevalence studies by coprology on fecal samples from 1,202 sheep and 2,690 cattle collected from different zones of the Northern Bolivian Altiplano. Prevalences were pronouncedly higher and more homogeneous in sheep (63.1%; range: 38.9–68.5%) than in cattle (20.6%; range: 8.2–43.3%) in each one of the different zones. Although similarities between the prevalences in sheep and cattle appeared in the zones of the highest and lowest infection rates, this disappeared in the other zones due to cattle treatments. Comparison with past surveys demonstrates that this hyperendemic area is stable from the disease transmission point of view. Therefore, the control design should prioritize sheep and cattle within the One Health action. Studies performed in the Bolivian Altiplano furnish a baseline for future initiatives to assess the transmission and epidemiological characteristics of fascioliasis in the way for its control in other high altitude Andean endemic areas.

## Introduction

Fascioliasis is a helminthiasis caused by two liver fluke species of the trematode genera *Fasciola, Fasciola hepatica*, and *Fasciola gigantica*, which infect herbivorous mammals and humans by ingestion of the infective stage of encysted metacercaria attached to freshwater plants but also floating metacercariae together with drinking water (1). After hatching in the small intestine, the juvenile worms cross the intestinal wall, migrate through the abdominal cavity until contacting with an hepatic lobe, and penetrate through the liver parenchyma up to finally reach the bile ducts where the adult stage will develop (2). The migratory phase by the juvenile worms is known as migratory or acute period of the disease, takes only a few months, and is characterized by a high pathogenicity and clinical impact (3, 4). The parasitation of the biliary passages and gallbladder by the adult stage is known as biliary or chronic period, may last several years, and has recently proved to also be able to produce severe pathological and clinical effects detectable by hematological and biochemical biomarker analyses (5, 6) and immunological assays (7). Fascioliasis is a well-known disease in livestock and causes very important economic losses in husbandry worldwide (8, 9). Its repercussions in public health have been the focus of many studies and surveys since the beginning of the 1990s (10), concerning not only developing countries but also developed countries (11). This worldwide complex scenario led the World Health Organization (WHO) to include fascioliasis within the main neglected tropical diseases list (12).

In the Americas, only *F. hepatica* is present. The absence of *F. gigantica* should be considered an advantage considering the epidemiological and pathological/clinical differences between both (13). In the New World, measures and interventions for the control of fascioliasis are consequently expected to be simpler. However, there is an aspect which differentiates human endemic areas in the Americas from those in other continents: the high altitude. Indeed, serious public health problems and human infection cases have been reported from rural areas and also neighboring villages and even towns at high and very high altitudes. It shall be considered that the term “very high altitude” is used to refer to altitudes higher than 3,200 m above sea level (a.s.l.). Human endemic areas have been reported in the Andean regions of countries such as Argentina (14–16), Bolivia (17–19), Peru (20–22), Ecuador (23), Venezuela (24), and Colombia (25) but also in the highlands of Mexico (26). Interestingly, even in Brazil, the highest human fascioliasis-positive area concerns Curitiba, at 1,000 m a.s.l. (27).

In the rural areas at high altitude, the extreme climate conditions pose problems to plant cultures and agricultural work and in most cases lead inhabitants to mainly depend on livestock. Moreover, in such remote areas there is usually lack or insufficiency of minimum supplies for human development, such as availability of potable water, electricity, and paved roads. The coexistence of both humans and livestock animals is ideal for a snail-borne zoonotic disease as fascioliasis (28).

Additionally, the aforementioned endemic areas are located in tropical and subtropical zones, which underlie climatic conditions, mainly temperature, allowing for the establishment of the specific freshwater lymnaeid snail vectors and the transmission of *F. hepatica* (29, 30). Indeed, liver fluke transmission is markedly dependent on meteorological factors (31). In this sense, both animal fascioliasis (32) and human fascioliasis (33) have already proved to be influenced by the climate change phenomenon. Even other climate phenomena, repetitive although not of periodic regularity, have been described to influence *F. hepatica* transmission, such as La Niña in South America (34).

The Northern Bolivian Altiplano, located at 3,820–4,100 m a.s.l., between Lake Titicaca and the valley of the capital city La Paz, is the hyperendemic area where the highest prevalences and intensities of fascioliasis have been reported in humans (28) and where children become infected very early in their lives, as observed in other human hyperendemic areas (35). In this area, each Aymara family traditionally sustains its own animals, usually more than one species, mainly sheep and cattle and secondarily pigs and donkeys. The increase of livestock has led to soil compaction by overgrazing, which in turn has progressively decreased agricultural soil capacity. Worth mentioning is that the mathematical models of fascioliasis commonly used in the Northern Hemisphere showed that liver fluke transmission was impossible under the extreme environmental conditions of the Northern Bolivian Altiplano (29). This poses questions about the disease transmission rates at such a very high altitude and the levels of contribution of each one of the aforementioned four animal species to this transmission.

The World Health Organization (WHO) launched a wide fascioliasis control initiative in this area in 2007–2008. Results of surveys in humans (36, 37) and interannual monitoring assessments in animals furnished an overall picture showing high infection and reinfection risks. Moreover, global warming has resulted in spread of *Galba truncatula*, the only lymnaeid species in these hyperendemic areas, and thus impacted on *F. hepatica* transmission (38).

Faced with this situation, the Pan American Health Organization (PAHO) organized a meeting in La Paz with the participation of the Bolivian Ministry of Health, the Ministry of “Desarrollo Rural y Tierras de Bolivia,” and the Servicio Departamental de Salud de La Paz, together with experts on human and animal fascioliasis, on November 10–12, 2014. In this meeting, the decision was taken to start a One Health action including long-term experimental studies, field monitoring assessments, and control activities with the aim of decreasing *F. hepatica* transmission and infection risks. This One Health initiative is at present ongoing and covers all multidisciplinary aspects of fascioliasis, according to modern standards (39–41). The purpose of the present study is to summarize the results obtained in experimental studies on *F. hepatica* transmission and field surveys on animal infection to assess the contribution to the disease epidemiology by the two main reservoir species sheep and cattle in the Northern Bolivian Altiplano, to furnish the needed baseline on which to design the appropriate control measures.

## Materials and Methods

### Experimental Assays

#### Liver Fluke Materials

**Figure 1:**
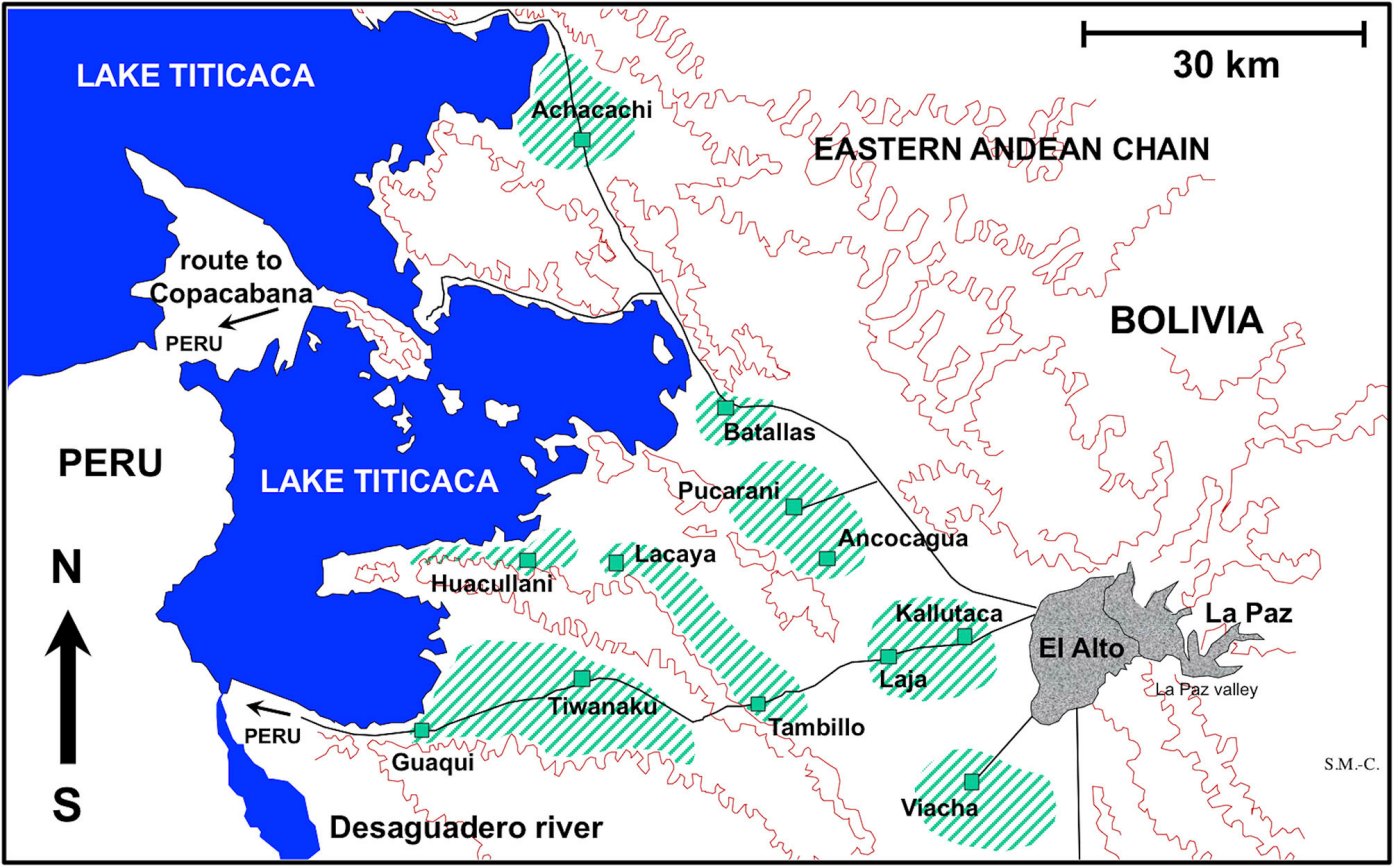
Map showing the Northern Bolivian Altiplano human fascioliasis hyperendemic area, at 3,820–4,100 m altitude, including zones where sheep and cattle were surveyed and localities where lymnaeid snail specimens of *Galba truncatula* were collected.

For the experimental study of the embryonation of the eggs of *F. hepatica*, fecal samples from three naturally infected sheep individuals from the locality of Batallas and from three naturally infected cattle from Kallutaca were used (Figure 1). Eggs isolated by filtration (filter pore size of 40 μm) were conserved in natural water under complete darkness at 4°C at very high altitude until starting of the embryogenesis follow-up study.

Given the irresolvable difficulties in performing the necessary experimental studies at the very high altitude in Bolivia due to the unavailability of the appropriate infrastructures (equipments, devices) and logistics (permanent functioning provisions, energy supplies, and security systems), an appropriate experimental approach was designed. Experiments were performed in a laboratory at low altitude. To minimize the influence of oxygen pressure (low at the very high altitude when compared to low altitude), experimental studies were started immediately after each field mission and arrival to the laboratory, i.e., altiplanic *F. hepatica* eggs were kept at low altitude only hours or maximum 1 and 2 days before beginning of egg embryonation, and only the first laboratory generation of altiplanic lymnaeids were used. Moreover, all life-cycle phases experimentally studied occur in freshwater, in which the difference of oxygen pressure is lower than in air.

For the experimental infection of lymnaeid snails, *F. hepatica* eggs were similarly obtained from sheep from Batallas and from cattle from Kallutaca and Batallas (Figure 1). Eggs were similarly isolated by filtration and conserved until used for snail infection in the laboratory. No distinction was made between isolates, which may be considered representative of the whole endemic area, as previous molecular studies have demonstrated the uniformity of parasite and snail involved in the disease throughout the whole Northern Bolivian Altiplano (38).

For the experimental infection of Wistar rats, *F. hepatica* metacercariae were obtained from the aforementioned experimentally infected lymnaeid snails. These metacercariae were stored in natural water in total darkness 4°C until required (42).

#### Egg Embryogenesis Follow-Up

Embryogenesis was microscopically followed at constant 20°C, with analytical observations every 4 days. This study was made in the climatic chambers of the Parasitology Department of the University of Valencia, Spain. Egg development was made by differentiation of (i) eggs in the phase of morula, showing vitelline granules and/or spheroidal cells (EM), (ii) eggs in the phase of outlined miracidium, in which a miracidial form begins to be observed (EOM), and (iii) eggs in the phase of developed miracidium, in which a fully developed miracidium is observed inside (EDM). For each 4-day study, a total of 33 eggs from each host individual were analyzed. Counting included not only EM, EOM, and EDM but also (i) degenerated eggs, (ii) empty eggs, and (iii) broken eggs. Egg counts were noted in percentages independently for sheep or cattle per observational day.

#### Miracidial Infection of Snails

Developed miracidia were forced to hatch by putting fully embryonated eggs under light and used for the experimental infection of snails (43, 44). Only laboratory-reared specimens were used. Lymnaeid snails of a size of 4–5 mm were used to assess infection susceptibility, by exposing each snail to miracidia for 4 h in a small Petri dish containing 2 ml of freshwater. Mono-, bi-, and trimiracidial infections were carried out. The disappearance of the miracidia was taken as verification of its successful penetration into the snail.

The sheep *F. hepatica* isolate was used for several infection assays under different experimental conditions concerning (i) miracidial dose and (ii) day/night temperature according to a photoperiod of 12-h light/12-h darkness in high-accuracy climatic chambers (HPS-1500, VB-0714, and HPS-500 models of Heraeus-Vötsch) (45). A similar procedure was followed with the cattle *F. hepatica* isolate. The characteristics, conditions, and number of snails in all these infection experiments are detailed in Table 1. After the infection, snails were returned to 2,000-ml containers, at 90% relative humidity (r.h.), 12/12 h light/darkness, and dry lettuce *ad libitum*, until day 30 post-infection. Afterward, they were again isolated in Petri dishes to allow daily monitoring of cercarial shedding by individual snails. Lettuce was provided *ad libitum* to each snail in a Petri dish during both shedding and post-shedding periods until death of the snail. The cercarial shedding was followed by daily counting of metacercariae in each Petri dish.

**Table 1:** Experimental infections of altiplanic lymnaeid snails with altiplanic sheep and cattle isolates of *Fasciola hepatica*.

**Host isolate**	**Sheep**			**Cattle**		
*F. hepatica* geogr. origin	Batallas	Batallas	Batallas	Batallas	Kallutaca	Kallutaca
Lymnaeid geogr. origin	Huacullani	Huacullani	Ancocagua	Huacullani	Viacha	Tambillo
Miracidial dose	Mono-miracidial	Mono-miracidial	Bimiracidial	Mono-miracidial	Mono-miracidial	Trimiracidial
Temperature (12 h day/12 h night)	20/20°C	22/5°C	20/20°C	20/20°C	22/5°C	20/20°C
No. of lymnaeids infected	62	50	37	55	21	50
No. of survivor snails at beginning of shedding (%)	54 (87.1%)	41 (82%)	13 (35.1%)	48 (87.3%)	13 (61.9%)	23 (46%)
No. of shedding snails (%)	28 (51.8%)	9 (21.9%)	7 (53.8%)	12 (25%)	2 (15.4%)	15 (65.2%)
Pre-patent period in dpi (mean)	48–92 (55.6)	59–93 (69.3)	41–69 (49.7)	49–76 (55.5)	94–95 (94.5)	43–95 (67.2)
Shedding end in dpi (mean)	52–136 (89.4)	70–93 (75.7)	49–87 (67.6)	58–135 (101.6)	95–106 (100.5)	70–117 (89.3)
Shedding length in days	1–88 (34.7)	1–15 (7.3)	6–29 (18.9)	1–85 (47.1)	1–13 (7)	1–85 (23.1)
No. of total cercariae shed	5,542	311	429	3,672	30	762
No. of cercariae/snail (mean)	8–562 (197.9)	1–155 (34.5)	15–101 (61.3)	8–581 (306)	1–29 (15)	2–100 (51.8)
Snail survival after shedding end in days	1–132 (24.5)	0–34 (5)	1–10 (3.9)	1–133 (42.3)	0–25 (12.5)	1–21 (3.8)
Longevity of shedding snails in dpi	53–192 (113.8)	72–127 (75.9)	51–90 (71.4)	76–268 (143.9)	106–120 (113)	71–128 (93.1)
Longevity of non-shedding snails in dpi	49–196 (139.1)	56–143 (83.2)	44–107 (62.7)	31–209 (105.4)	96–161 (132.9)	32–49 (38.5)

dpi, days post-infection.

#### Lymnaeid Snail Laboratory Cultures

The presence of only *G. truncatula* as only lymnaeid species inhabiting the Northern Bolivian Altiplano hyperendemic area has recently been confirmed by the sequencing of complete nuclear ribosomal DNA and mitochondrial DNA markers (38). This species is of European origin and differs from the Neotropical species of the *Galba*/*Fossaria* group of lymnaeids which also act as vectors of fascioliasis in South America (46). Live *G. truncatula* snails were collected in the localities of Huacullani, Batallas, Tambillo, and Viacha (Figure 1) and transported under isothermal conditions for their laboratory adaptation in Valencia to standardized controlled conditions of 20°C, 90% relative humidity, and a 12/12-h light/darkness photoperiod in the aforementioned precision climatic chambers. The possible natural infection by fasciolids was always individually verified prior to the launch of laboratory cultures. This was performed by keeping each lymnaeid snail isolated in a Petri dish containing a small amount of natural water. After 24 h, the presence or absence of motionless metacercarial cysts or moving cercariae was verified in each Petri dish. Non-infected lymnaeids were arranged in standard breeding boxes containing 2,000 ml freshwater, to assure locality-pure cultures. The water was changed weekly and lettuce added *ad libitum*.

#### Infections of Laboratory Mammal Model

A total of 45 male Wistar rats (Iffa Credo, Barcelona, Spain) aged 4–5 weeks were used throughout. A balanced commercial rodent diet (Panlab Chow A04) and water were provided *ad libitum*, according to standards previously described (47).

Wistar rats were infected according to methods previously described (48). Metacercariae were inoculated orally by means of a gastric tube. A dose of 20 *F. hepatica* metacercariae per rat was used (Table 2). Animal care, animal health, body condition, and well-being were assessed on a weekly basis by means of checking their body weight and the appearance of the fur. Infected animals presented a lower body weight than negative controls at the end of the experiment. No mortality occurred. The number of Wistar rats in each infection experiment is noted in Table 2. Finally, animals were humanely euthanized with an overdose of an anesthetic (IsoFlo; Dr Esteve SA, Barcelona, Spain), and *F. hepatica* worms were collected under a dissecting microscope, according to methods already outlined before (49). Infection prevalence and intensity (number of worms successfully developed in each rat) were established by necropsy 12 weeks after infection. Initially, the bile duct was examined for the presence of worms, though the rest of the organs were also evaluated. Afterward, the thoracic and abdominal viscera and cavities were examined and thoroughly rinsed with water to assure the recovery of all worms. After the analysis of the definitive host infectivity of the sheep isolate in a sufficient number of experimentally infected rats, and considering present knowledge indicating the absence of infectivity differences between sheep and cattle isolates, in agreement with present ethical recommendations, a minimum number of rats were used to assess this aspect in the cattle isolate.

**Table 2:** Experimental infections of Wistar rats with experimentally obtained *Fasciola hepatica* metacercariae from sheep and cattle isolates from the Northern Bolivian Altiplano human hyperendemic area.

**Host isolate**	**Sheep**		**Cattle**	
*F. hepatica* geographical origin	Batallas	Ancocagua	Kallutaca	Batallas
Age of metacercariae	1 week	2 weeks	6 weeks	8 weeks
No. of metacercariae inoculated per rat	20	20	20	20
No. of inoculated rats	14	23	4	4
No. of rats infected (%)	11 (78.6%)	18 (78.3%)	4 (100%)	2 (50%)
No. of flukes recovered per rat (mean)	1–8 (3.6)	1–10 (3.7)	1–2 (1.7)	1–2 (1.5)
Intensity^a^	14.3%	14.6%	8.8%	3.7%
Mean % flukes recovered/rat^b^	18.2%	18.6%	8.8%	7.5%

a *Intensity = total % of flukes recovered = (total no. of flukes recovered/total no. of metacercariae administered in all rats) × 100*.

b *Mean % flukes recovered/rat = Mean % of flukes recovered per infected rat = (flukes recovered/metacercariae administered per infected rat) × 100*.

### Field Surveys of Sheep and Cattle

#### Host Animals Studied

Fecal samples from a total of 1,202 sheep and 2,690 cattle were collected from different zones of the Northern Bolivian Altiplano (Table 3). Ovines of the Corriedale, Merino, Criollo, and Mestizo breeds (Mestizo = crossbreeds Criollo × Corriedale or Criollo × Merino), aged 1 month−6 years, and bovines of the Holstein, Brown Swiss, native, and crossbreeds aged 5 months−12 years were included. The geographical distribution of the zones surveyed is shown in Figure 1.

**Table 3:** *Fasciola hepatica* prevalence and intensity found by coprological analyses in sheep and cattle of different zones of the Northern Bolivian Altiplano human hyperendemic area.

**Host species**	**Sheep**			**Cattle**		
**Endemic zones**	**No. analyzed/no. infected**	**Prevalence (%)**	**Intensity range (mean) in epg**	**No. analyzed/no. infected**	**Prevalence (%)**	**Intensity range (mean) in epg**
Achacachi	252/145	57.5^a^	12–241 (96.5)	515/147	28.5	3–96 (12.6)
Batallas	135/76	56.3^a^	9–145 (70.1)	303/45	14.8	1–49 (7.2)
Pucarani	113/44	38.9^a^	3–9 (4.2)	426/35	8.2	1–30 (4.5)
Kallutaca-Laja	117/69	58.9^a^	6–27 (23.2)	379/121	31.9	3–88 (5.9)
Tambillo-Lacaya	204/137	67.1^a^	3–21 (10.4)	141/17	12	1–20 (5.2)
Huacullani	296/203	68.5^a^	6–35 (25.9)	196/85	43.3	1–20 (7.2)
Guaqui-Tiwanaku	85/45	52.9^a^	3–12 (9.8)	352/31	8.8	1–24 (4.3)
Viacha	–^**b**^	–^**b**^	–^**b**^	381/773	19.1	1–93 (4.5)
Extreme values	85–296/44–203	38.9–68.5	3–241	141–515/17–147	8.2–43.3	1–96
Mean	171.7/108.4	57.1	54.7	336.2/69.2	20.8	6.5
Total	1,202/759	63.1^a^		2,690/554	20.6	

a *Significantly different vs. cattle, determined by chi-square test (P < 0.05)*.

b *No sheep studied from this zone*.

The surveys were conducted at random. All animals that were available to us were sampled in each zone, to assure a representative sample size. The zones studied cover all the flatlands, in between the hilly chains, throughout which the disease is detected affecting humans and lymnaeid vectors involved in the transmission are present (28, 38). The term of “herd” can be hardly applied to the Northern Bolivian Altiplano, except to the large flatlands of the farms of Achacachi, Kallutaca, and Viacha where no transmission to humans has been reported. In the human endemic zones, Aymara inhabitants used to own a very few animals per family, if any, and these animals are not managed as herds but mostly allowed to run free, only rarely as isolated individuals kept in place by stake and rope. Therefore, the infection rates in sheep and cattle were analyzed according to geographical zones instead of to herds.

Moreover, it should be considered that treatments of animals, mainly cattle, are regular and programmed in the zones of Achacachi, Kallutaca, and Viacha, whereas in other zones the animals are irregularly treated (when animals appear to be affected or when the owner had funds to treat them) or not treated at all.

#### Fecal Sample Preparation and Study

Only coprological methods for qualitative and quantitative analyses were carried out. Fecal samples were placed in numbered plastic bags, transported to the laboratory of La Paz within the following 5 h, and maintained in a fridge at 4°C until examination. From each fecal sample, a quantity of 4 g was sedimented twice, first with 50 ml of detergent solution (1 ml/1,000 cm^2^) after filtration and second with 50 ml water, and stained with methyl green according to a standard method (50), before examination under light microscope for *F. hepatica* eggs. A sample was considered negative when no eggs were found in its respective fecal sample after studying 3 slides. The number of eggs shed by a sheep or cattle was used to estimate the infection intensity and was expressed in eggs per gram of feces (epg). For this purpose, the whole sediment was microscopically analyzed in the positive samples, and the number of fluke eggs found was divided by 4 to obtain the epg value.

### Statistical Analyses

Statistical analysis was performed using SPSS Statistics version 26. Development egg data (EM, EOM, and EDM) in sheep and cattle isolates were compared by ANOVA test. Statistical comparison of categorical variables was carried out with the Chi-square test and Yates continuity corrected Chi-square test. Means obtained in data from experimental infections of lymnaeid snails were compared by *t*-test. Means obtained in data from experimental infections of Wistar rats were compared by non-parametric Mann–Whitney test. Results were considered statistically significant when *P* < 0.05.

## Results

### Egg Embryonation

**Figure 2:**
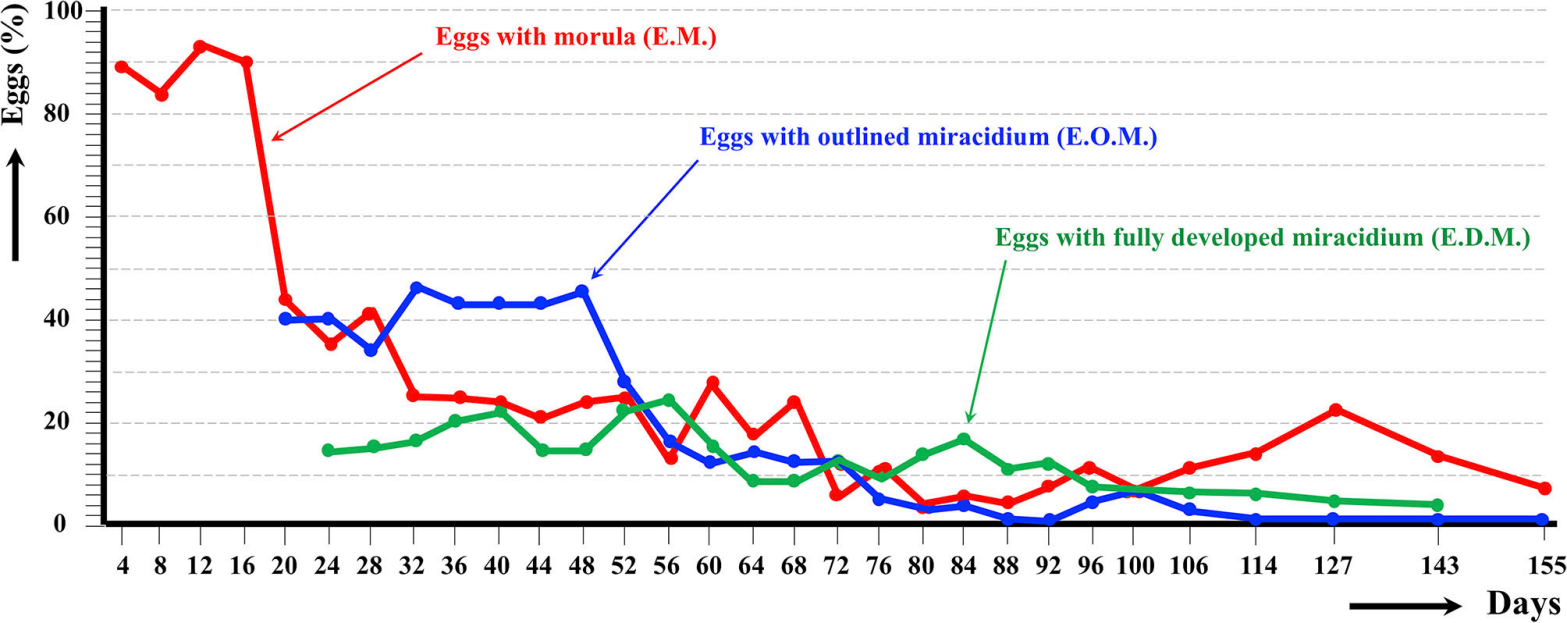
Results of the experimental follow-up study of the egg embryonation of the altiplanic sheep isolate of *Fasciola hepatica*, at 4-day study intervals and constant temperature of 20°C. Percentages of degenerated, empty, and broken eggs are not included.

**Figure 3:**
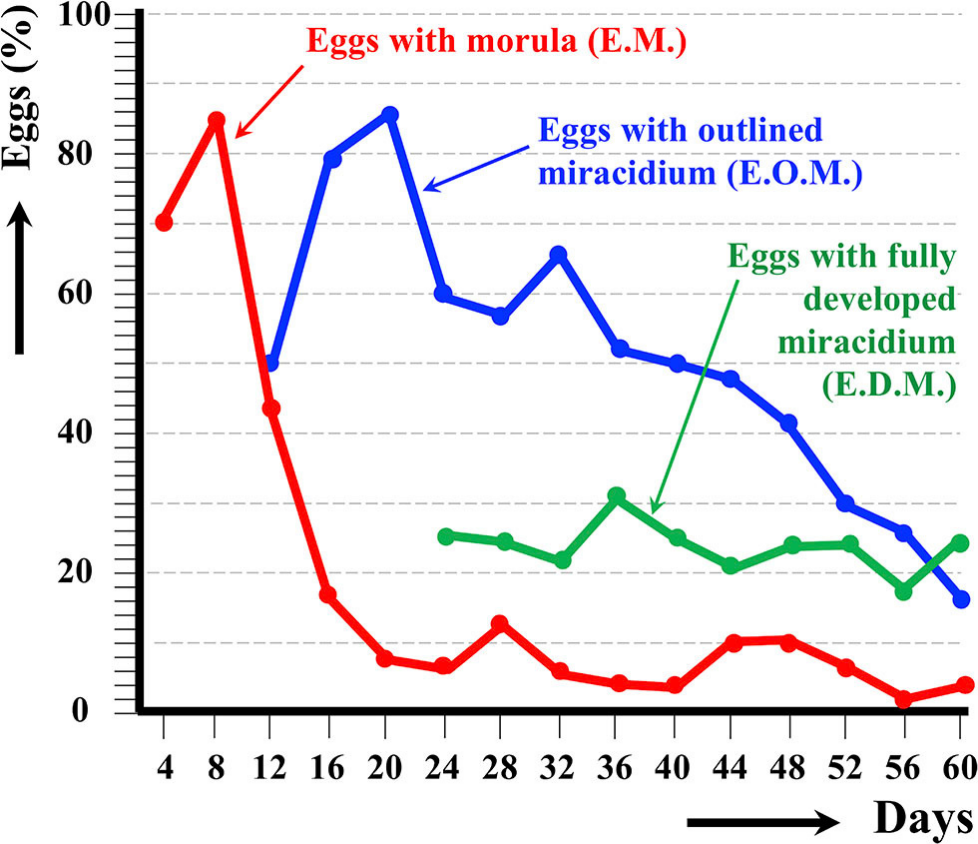
Results of the experimental follow-up study of the egg embryonation of the altiplanic cattle isolate of *Fasciola hepatica*, at 4-day study intervals and constant temperature of 20°C. Percentages of degenerated, empty, and broken eggs are not included.

The results of the experimental follow-up of the egg embryonation of the sheep and cattle isolates of altiplanic *F. hepatica* are shown in Figures 2, 3, respectively. Degenerated, empty, and broken eggs (eggs easily break when open and empty after miracidial release) are very few at the beginning but of course increase with time and are the marked majority in the last days of the follow-up study. Percentages of degenerated, empty, and broken eggs are not included in the graph of follow-up curves because they do intervene in the transmission.

An outlined miracidium form begins to be observed inside eggs at 20 and 12 days, respectively, and the first fully developed miracidium already appears at day 24 in both isolates. Eggs including a fully developed miracidium were henceforth observed in each observational day until 143 and 60 days, respectively. Days in which analyzed eggs showed the highest percentage of fully developed miracidia were 56 and 36 days, respectively. The average egg embryonation (EOM, EDM) in the 60 days showed higher values (*P* = 0.015) in sheep isolate (38.93%) than in cattle isolate (26.63%).

### Lymnaeid Infectivity and Intramolluscan Development

Results of the different experimental lymnaeid infection assays performed with the sheep and cattle isolates are shown in Table 1. No statistically significant differences (*P* = 0.9941) were found in snail infectivity, assessed by the percentage of snails successfully infected by a monomiracidial dose at 20/20°C in the sheep isolate and cattle isolate, although snail infectivity was higher in the sheep isolate under temperature conditions of both 20/20°C and 22/5°C. However, the result was the opposite in the assays performed at 20/20°C when comparing the infectivity of a bimiracidial dose in the sheep isolate with that of a trimiracidial dose in the cattle isolate.

The pre-patent period in monomiracidial infections was similar in both isolates both at 20/20°C and at 22/5°C, and no statistically significant differences (*P* = 0.0699) were found in the values of the pre-patent period. This period was shorter when bimiracidially infecting with the sheep isolate (*P* = 0.1039) than in the trimiracidial infections with the cattle isolate (*P* = 0.4661), although no statistically significant differences appeared. The cercarial shedding period was similarly long in all assays when comparing sheep with cattle isolates, as confirmed by the absence of statistically significant differences (*P* = 0.0998).

Regarding the production of cercariae per infected snail, results were similar between the two altiplanic *F. hepatica* isolates, and no statistically significant differences (*P* = 0.2212) were found. However, the cattle isolate monomiracidial infection assay cannot be considered because of the very low number of successfully infected lymnaeids.

There were surprisingly long snail survivals after shedding ended. This aspect did not show significant differences between both isolates, although bimiracidial and trimiracidial infections appear to reduce lymnaeid survival. When comparing the longevity of successfully infected snails with that of the non-infected ones, the absence of differences is worth mentioning.

### Definitive Host Infectivity

Results of the experimental infection of Wistar rats with experimentally obtained metacercariae of the altiplanic sheep and cattle isolates are shown in Table 2. No infectivity differences appear between the two isolates concerning the percentage of rats successfully infected (sheep: 78.4%; cattle: 75%; *P* = 0.9999). The intensity of infection, measured by the number of flukes recovered per metacercariae inoculated and also per rat, however shows differences, although the number of rats inoculated was unfortunately too low as to reach a significant conclusion (sheep: 18.5%; cattle: 8.4%; *P* = 0.9999).

### Natural Infection Rates

Results of the coprological surveys on sheep and cattle populations inhabiting eight selected zones of the fascioliasis hyperendemic area are shown in Table 3. Unfortunately, sampling could not be made in the different seasons and therefore prevalence variation throughout the year could not be analyzed. Prevalences were pronouncedly higher in sheep than in cattle in each one of the different zones. Although there is a similarity between the prevalences in sheep and cattle in both the zones of the highest (Huacullani) and lowest (Pucarani) infection rates, this disappears in the other zones.

In sheep, prevalences found were high and very homogeneous, with a total prevalence of 63.1% and a range of 38.9–68.5% according to zones. Among the different sheep breeds studied, there appears to be a higher susceptibility for *F. hepatica* infection in the Corriedale breed (83.2% prevalence) when compared to Merino, Criollo, and Mestizo (=crossbreeds Criollo × Corriedale or Criollo × Merino) (56.2–63.9%). Sheep aged <4 months appear to be the most infected (87.1%), whereas the prevalence decreases relatively with age, up to the 2–6-year-old group (56.9%).

In cattle, the total prevalence found in the Northern Bolivian Altiplano was 20.6%, significantly lower than the total prevalence found in sheep (*P* = 0.0001). The prevalences were irregularly distributed, varying considerably according to zones (8.2–43.3%). Cattle aged <1 year proved to be infected only sporadically (9.4%). The liver fluke prevalence increases pronouncedly in bovines aged 1–2 years (37.5%) and appears to be maintained at a similar level in animals aged more than 2 years (28.7%). No conclusion about potential susceptibility differences according to cattle breeds could be obtained, as the Brown Swiss in the Achacachi zone are regularly treated, similarly as the Holstein breed in Kallutaca and Viacha. The Creole breed, distributed throughout the remaining zones, is irregularly treated depending on owners or not treated at all.

Results on intensities in epg are noted in Table 3. No statistical comparison of intensities between sheep and cattle is included, because they are not comparable due to the much larger quantities of feces per day produced by cattle, and the consequent higher dilution of epg in this species regarding sheep.

## Discussion

### Egg Embryonation in Altiplanic Sheep and Cattle Isolates

The environment of the high altitude is characterized by (i) decreases in oxygen and air density, (ii) low temperature and humidity, and (iii) an increase in solar radiation. These environmental factors exert an influence on the animals and humans born and living at high altitude (51, 52). Thus, livestock inhabiting high-altitude zones exhibit several changes, such as hypoxia (53), alterations in immune response (54), elevated hematocrit levels, differences in blood oxygen pressure values and blood viscosity (55), and elimination of dissolved gases, especially N_2_, from the blood (56).

These changes may influence the development of *F. hepatica*, mainly because of its tissue migration and hematophagous diet (57, 58). Moreover, the miracidium of *F. hepatica* within the egg is stimulated by high oxygen tension. At different oxygen tensions, the *F. hepatica* eggs showed little variation in mortality, but those in aerobic conditions hatched in one-fifth of the time taken by those at lower oxygen tension (59). Indeed, *F. hepatica* miracidia are dependent on aerobic degradation of their endogenous glycogen stores by glycolysis and on Krebs cycle activity for energy generation. Studies proved that miracidia cannot function anaerobically, as inhibition of the respiratory chain blocks motility and carbohydrate degradation and finally results in death of the miracidia (60).

Little is known about the high-altitude impact on the development of parasites, including *F. hepatica*. In the Northern Bolivian Altiplano, no differences in egg size were found, and in adults only a smaller size in the majority of the parameters in the Bolivian specimens was found when compared with liver flukes from lowlands, as in Europe (61). However, liver fluke populations infecting altiplanic sheep and cattle proved to have a uterus significantly smaller than that in European lowlands (62). Moreover, a direct relation between *F. hepatica* uterus size and egg production (=number of eggs shed per g of feces) was experimentally demonstrated (63). It is known that oxygen is still required for egg production (64). Thus, high-altitude hypoxia conditions could be the origin of a reduced egg production by the flukes. In digeneans, the uterus is an organ adapted to the developmental time of the eggs (in fasciolids, eggs are laid unembryonated, the miracidium beginning its development in eggs once in freshwater). In the Northern Bolivian Altiplano, climatic conditions (29), together with the characteristics of freshwater collections and lymnaeid ecology (28, 38), enable *F. hepatica* transmission to take place throughout the year, so that egg storage in the uterus is less needed than in the northern hemisphere lowlands where *F. hepatica* transmission is typically seasonal.

Concerning the experimental studies performed, it should be considered that *F. hepatica* is able to readapt to different conditions, but this does not occur from one generation to the following one, but needs several generations for such changes and this means many years in nature. Consequently, it may be considered that the phenotypic characteristic of high-altitude adaptation was kept along all experiments.

The follow-up of the egg embryonation of the sheep and cattle isolates of altiplanic *F. hepatica* fit well in the present knowledge. Studies on *F. hepatica* egg embryonation are numerous, but unfortunately experimental conditions used are very different depending on the studies. A minimum temperature threshold of 10°C for the development of the egg of *F. hepatica* was established early on (65). The maximum temperature threshold was later established to be at 37°C, and the optimum temperature at 25°C (66). At 10°C, the time needed to reach 50% of the eggs presenting a fully developed miracidium inside was 161 days (59). In another study in southern Europe, the following hatching times were found: 56 days at 15°C, 50 days at 18°C, 27 days at 20°C, 17 days at 25°C, 22 days at 27°C, and 17.5 days at 30°C (67). In the southern part of Chile, results on first hatching times proved to be different: 101 days at 9.1°C, 80 days at 10°C, 57 days at 12.4°C, 44 days at 12.6°C, 42 days at 13.8°C, 34 days at 15.1°C, 28 days at 16°C, 30 days at 16.4°C, and 20 days at 17°C (68). When analyzing the linear correlation between egg development and temperature, a hatching time of 19–20 days was obtained for 20°C (69). So, the appearance of the first fully developed miracidium at day 24 in both altiplanic sheep and cattle isolates fits inside the 19–27-days range from the data of the aforementioned studies. This does not suggest a negative influence of the high-altitude environmental conditions on the *F. hepatica* egg embryonation.

### Miracidial Infectivity and Intramolluscan Development

Although knowledge on the infectivity rates of *F. hepatica* concerning snail infectivity, intramolluscan prepatent and patent periods, cercarial/metacercarial production capacity, and lymnaeid infection survival in endemic areas at very high altitude is lacking, there have been many experimental studies on the different aspects involved in these crucial steps of the parasite transmission in lowland areas. The species pair *F. hepatica*/*G. truncatula*, both from low altitudes of Europe, has been the most used for such experimental studies. Results indicate these processes to be highly complex, involving many different factors underlying a large variability between different populations of *G. truncatula*, different *G. truncatula* specimens within the same population, and even differences appearing in the same *G. truncatula* populations over time. Our experiments used *F. hepatica* from different altiplanic localities, with different miracidial infection doses, and at different daily evolving temperature (Table 1), to get the most accurate picture.

The infectivity (% of cercariae-shedding snails) of 51.8 and 25% monomiracidially obtained at 20/20°C with the altiplanic sheep and cattle isolates, respectively, fit the ranges of 32–57% and 14–56.8% experimentally detected in different *G. truncatula* populations from lowlands in France (70, 71). The quality of the furnished food is the main factor underlying such a wide variability (72). The higher infectivity rates found in the assays using bimiracidial and trimiracidial doses also agree with the present knowledge indicating a direct relationship between infectivity and miracidial dose (73). The 22/5°C day/night photoperiod assayed to reproduce the daily temperature variation in the Northern Bolivian Altiplano cannot, unfortunately, be compared because there are no previous studies on the influence of such drastically changing day/night temperature on *G. truncatula* infection by *F. hepatica* miracidia. The results of 21.9 and 15.4% monomiracidially obtained with the altiplanic sheep and cattle isolates should be emphasized. Indeed, these results indicate that a night temperature markedly below the minimum temperature threshold for *F. hepatica* does not impede the complete intramolluscan development. The lower infectivity percentages suggest that daily temperature variations are the cause of lower lymnaeid infection rates.

Experimental studies on *F. hepatica*/*G. truncatula* demonstrated that the pre-patent period (from infection up to the shedding of the first cercaria) is dependent on temperature, higher temperatures reducing its length: 56–86 days at 15°C, 48–51 days at 20°C, and 38 days at 25°C (66, 69, 74). The pre-patent periods shown by the altiplanic sheep and cattle isolates in mono- and trimiracidial infections at 20/20°C are therefore slightly longer than in the lowlands of Europe, but those at 22/5°C in the sheep isolate fit well within the range at 15°C (the too low number of successfully infected snails with the cattle isolate does not allow to reach any conclusion).

The patent or cercarial shedding period is also known to show large variability. At 20°C, different mean lengths were experimentally found in monomiracidial infections of *F. hepatica*/*G. truncatula* in the European lowlands: 18.6–28 days in cattle isolate (70) and 47.3 and 60.4 days in sheep and cattle isolates, respectively (75). Results of the monomiracidial infections with the altiplanic isolates at 20/20°C do therefore fit well within these ranges. The length of this patent period appears markedly reduced at 22/5°C. A reduction of the patent period is also observed in bi- and trimiracidial infections at 20/20°C, which agrees with results of previous studies (76).

### Cercarial Production, Lymnaeid Survival, and Metacercarial Infectivity

Results of cercarial productions per snail obtained in both altiplanic sheep and cattle isolates of *F. hepatica* in monomiracidial infections at 20/20°C (Table 1) are higher than those obtained in *F. hepatica*/*G. truncatula* in given populations from lowlands: 120 cercariae/snail (77) and 91.7 cercariae/snail (78). There are however those similar to productions found in other studies, such as 300 cercariae/snail (69), but lower than the range of 476–544 cercariae/snail (66) and far from the range of 952–2,275 cercariae/snail obtained when infected with many miracidia and providing special food (72).

The aforementioned numbers of cercariae/snail obtained with the altiplanic sheep and cattle isolates in monomiracidial infections at 20/20°C are pronouncedly higher than those obtained in the assays when under the conditions of 22/5°C and also in the respective assays with bi- and trimiracidial infection doses (Table 1). Concerning the influence of the number of miracidia used for the infections, previous studies with the same fluke/snail species pair indicate that bimiracidial infections furnish slightly lower cercarial productions than monomiracidial ones (73).

Survival rates in successfully infected altiplanic *G. truncatula* snails are worth mentioning. This does not only concern the longevity of shedding snails in days post-infection when compared to the longevity of the snails whose experimental miracidial infection failed in the same experiments but also concern the survival of the successfully infected specimens after the end of the cercarial shedding process. These results indicate a longevity pronouncedly longer in infected lymnaeids from high-altitude areas than the same survival period known in *F. hepatica*/*G. truncatula* in the lowlands and confirm early findings and analyses about this phenomenon which appears to be independent from the host isolate of *F. hepatica* (79).

Laboratory infections of Wistar rats with the experimentally obtained metacercariae allowed to verify the infectivity of the definitive host by the altiplanic sheep and cattle isolates used, including prevalences and intensities (Table 2) which fit well in the present knowledge when dealing with metacercariae of a short age (42, 80). Moreover, previous studies have already shown that no infectivity differences are found between metacercariae from sheep and cattle isolates (81, 82). Indeed, similar results were already obtained with metacercariae from the Northern Bolivian Altiplano (83).

### *Fasciola hepatica* Infection Rates in Altiplanic Sheep and Cattle

The total number of liver fluke eggs expelled with feces by a sheep or cattle per day in the Northern Bolivian Altiplano, according to the respective intensities detected (Table 3) and the amount of feces produced by an infected animal of these species per day (according to standard data: 1–3 kg feces/day in sheep; 15–35 kg feces/day in cattle), may be estimated between 3,000 and 723,000 eggs/sheep/day (mean 109,400 eggs/sheep/day) and between 15,000 and 3,360,000 eggs/cattle/day (mean 162,500 eggs/cattle/day). Despite this higher capacity of cattle to contribute egg contamination of the environment, several aspects indicate that sheep play a crucial role in fascioliasis transmission in the Altiplano.

First, concerning the way Aymara people inhabiting the endemic area manage their animals, it should be considered that there are no livestock populations isolated by fences throughout the human hyperendemic area. Sheep and cattle appear free everywhere, and only in a few places may animals be kept in place by stake and rope. However, sheep herds are usually moved from one place to another by women and/or children as in the Viacha farming zone—see illustration in Figure 10E, page 1,681, in the previous article (1), whereas such cattle movements are sporadic or by means of trucks—see illustration in Figure 5E, page 12, in the previous article (38).

Second, Aymara inhabitants give nowadays more importance to their cattle than to their sheep (84, 85). Altiplanic Aymara mostly focus anti-fasciolid treatment efforts on cattle. Indeed, treatment campaigns are regular over time in many cattle populations in the zones such as Achacachi, Kallutaca, and Viacha where very wide flatlands are used as “farms,” including treatments once or twice per year. In the remaining zones of Batallas, Tambillo-Lacaya, and Guaqui-Tiwanaku, family owners self-treat their cattle at irregular intervals depending on the individual availability of funds for the purchase of the needed drug. Triclabendazole is the drug currently used. On the contrary, sheep may only secondarily be treated in the aforementioned “farms,” but they are never or very seldom treated by the family owners. This is very important, because the geographic distribution of human infection risk in the Altiplano does not concern these “farms,” but the wide flatland zones along which very numerous dispersed human communities are present, people develop their daily activities, and children walk from home to school and back (28, 38). The significant differences in the prevalences between sheep and cattle according to the several zones studied (Table 3) may most probably also underlie differences in anti-fasciolid treatment efforts.

A third aspect concerns the natural immune response in cattle, which may underlie the prevalences which do not increase in animals aged more than 2 years found in the Altiplano. As it has been emphasized, in cattle the disease is self-limiting, most of the flukes are eliminated within 9–12 months, the duration of egg production is short and high egg output lasts for only a few weeks, and resistance is acquired during primary infection (86). Interestingly, nucleotide polymorphisms in given regions of the bovine genome have been recently found in cattle presenting resistance to *F. hepatica* (87). A shorter life span of the liver fluke and a shorter egg shedding in cattle infections are opposite to sheep infections in which the fasciolid adult stage may reach 11 years showing continuous egg production (86).

The fourth aspect to be considered here is the high monomiracidial infectivity of the sheep isolate, which is more than double that of the cattle isolate (Table 1). A broader capacity of snail infection assures a wider diffusion of metacercariae in the environment and hence higher human and livestock infection probabilities.

The analysis according to zones is the most convenient from the One Health control perspective, because it allows for geographical comparisons with respective human infection rates. When comparing with the results of surveys on liver fluke infection in sheep and cattle in the Northern Bolivian Altiplano hyperendemic area made in the past (28, 88, 89), infection rates found in the present study appear to be very similar and indicate a long-term stable endemic situation. The higher prevalences in cattle found in the past surveys may probably be due to increased treatments in the recent years.

## Concluding Remarks

The experimental studies performed on the development stages of egg, miracidium, lymnaeid snail infection, intramolluscan larval development, cercarial production, snail survival to infection, metacercarial infectivity of mammal host, and adult stage development demonstrate that the *F. hepatica* isolates from altiplanic sheep and cattle show the capacity to assure the transmission of *F. hepatica* in the very high-altitude hyperendemic area of the Northern Bolivian Altiplano by their own, independently one isolate from the other. The level of transmission provided by both isolates fits in the ranges known in the same transmission aspects in lowland areas of Europe, where the transmission and epidemiology of the disease pronouncedly differ from the Andean highlands of South America (44). Moreover, under the extreme day/night temperature conditions of this area, the aforementioned transmission aspects also appear to be able to be sufficient, despite their lower efficiency.

The results of the field surveys on liver fluke infection rates in sheep and cattle populations inhabiting the different zones of the Northern Bolivian Altiplano and their comparison with similar field data obtained on the same livestock species in past surveys in the same zones demonstrate that this hyperendemic area is stable from the disease transmission point of view.

The results obtained indicate that altiplanic sheep and cattle should be considered the main animal reservoir species involved in *F. hepatica* transmission and therefore also of the infection and reinfection risks of humans and which have immunological and clinical consequences (90, 91). The infections and reinfections in children are detected in the monitorings by appropriate coprological techniques (37, 92) in between yearly mass treatment campaigns implemented in this hyperendemic area.

Samples from animals were obtained in the close proximity of human communities or households. It should be considered that (i) the Aymara concept of village or community usually concerns markedly dispersed dwellings which allow the animals to walk in between and around, (ii) 5–15-year-old children living in small communities not provided with a school are in need for a daily walk along rural unpaved roads to the nearest school which in many cases may be relatively distant, (iii) freshwater collections inhabited by lymnaeids and grazing free sheep and cattle used to be present in the neighborhood or besides these rural ways, and (iv) such home–school–home walks have been verified to represent a high infection risk for the children (1).

Consequently, sheep and cattle should be considered priority within the One Health action, including management measures to decrease their infection probabilities and appropriate treatments of the infected animals to decrease field contamination and lymnaeid infection rates. Drugs different from triclabendazole, such as closantel, should be used to treat these animals, to avoid the appearance of a potential resistance in *F. hepatica* which could give rise to problems in the preventive chemotherapy campaigns for humans, which are based on Egaten® (triclabendazole for human use).

Studies performed in the Bolivian Altiplano furnish a useful baseline for future similar initiatives to assess the transmission and epidemiological characteristics of fascioliasis in the way for its control at high altitude. It should however be considered that, in Andean endemic areas, fascioliasis follows a different transmission pattern in valleys and in the Altiplano (93) and that the new *F. hepatica* coproantigen detection techniques may facilitate the task in ruminants (94), similarly as in humans.

## Data Availability Statement

All datasets generated for this study are included in the article/supplementary material.

## Ethics Statement

All experimental research was performed with the approval of the Evaluation of Projects concerning Animal Research at University of Valencia (Organo Habilitado para la Evaluación de Proyectos de Experimentación Animal de la Universidad de Valencia) (A1263 915389140), strictly following the institution's guidelines based on Directive 2010/63/EU. Permission for animal research was additionally obtained from the Servicio de Sanidad y Bienestar Animal, Dirección General de Producción Agraria y Ganadería, Consellería de Presidencia y Agricultura, Pesca, Alimentación y Agua, Generalitat Valenciana, Valencia, Spain (No. 2015/VSC/PEA/00001 tipo 2). Animal ethics guidelines regarding animal care were strictly adhered. The study was approved by the Comisión de Etica de la Investigación of the Comité Nacional de Bioética, La Paz (Certificate dated 10 September 2007), Comité de Etica y Bioética de la Facultad de Medicina de la Universidad Mayor de San Andres, UMSA, La Paz—COMETICA (Resolución COMETICA No. 03/2019, dated 23 July 2019), and Comité de Revisión Etica (PAHOERC) of the Pan American Health Organization, PAHO, Washington DC (Dictamen Ref. No. 2018-02-0007, dated 10 September 2019). All investigations were made after permission was obtained from local Aymara Community Chiefs (Jilakatas and Malkus), and with the consent of animal owners.

## Author Contributions

SM-C performed One Health studies, designed the protocols, wrote the manuscript, and obtained project funding. PB performed the surveys and diagnosis of animals. IRF performed the snail cultures and experimental snail infections. RA coordinated local research and logistics and participated in surveys. PA participated in surveys and diagnosis of animals. MAV performed the experimental mammal infections and statistical analyses and obtained project funding. MDB performed the egg development studies, designed and participated in the experimental snail infections, participated in surveys, and obtained project funding. All authors contributed to the article and approved the submitted version.

## Conflict of Interest

The authors declare that the research was conducted in the absence of any commercial or financial relationships that could be construed as a potential conflict of interest.

## References

[1] Mas-Coma S Bargues MD Valero MA Human fascioliasis infection sources, their diversity, incidence factors, analytical methods and prevention measures. Parasitology. (2018) 145:1665–99. 10.1017/S0031182018000914 29991363

[2] Gonzalez-Miguel J Valero MA Reguera-Gomez M Mas-Bargues C Bargues MD Simon-Martin F. Numerous *Fasciola* plasminogen-binding proteins may underlie blood-brain barrier leakage and explain neurological disorder complexity and heterogeneity in the acute and chronic phases of human fascioliasis. Parasitology. (2019) 146:284–98. 10.1017/S0031182018001464 30246668 PMC6402360

[3] Chen MG Mott KE Progress in assessment of morbidity due to *Fasciola hepatica* infection: a review of recent literature. Trop Dis Bull. (1990) 87:R1–38.

[4] Mas-Coma S Agramunt VH Valero MA. Neurological and ocular fascioliasis in humans. Adv. Parasitol. (2014) 84:27–149. 10.1016/B978-0-12-800099-1.00002-8 24480313

[5] Valero MA Navarro M Garcia-Bodelon MA Marcilla A Morales M Garcia JE. High risk of bacterobilia in advanced experimental chronic fasciolosis. Acta Trop. (2006) 100:17–23. 10.1016/j.actatropica.2006.09.002 17064656

[6] Valero MA Girones N Garcia-Bodelon MA Periago MV Chico-Calero I Khoubbane M. Anaemia in advanced chronic fasciolosis. Acta Trop. (2008) 108:35–43. 10.1016/j.actatropica.2008.08.007 18805388

[7] Girones N Valero MA Garcia-Bodelon MA Chico-Calero MI Punzon C Fresno M. Immune supression in advanced chronic fascioliasis: an experimental study in a rat model. J Infect Dis. (2007) 195:1504–12. 10.1086/514822 17436231

[8] Copeman DB Copland RS Importance and potential impact of liver fluke in cattle and buffalo. In: Gray GD, Copland RS, Copeman DB, editors. Overcoming Liver Fluke as a Constraint to Ruminant Production in South-East Asia. Canberra: Australian Centre for International Agricultural Research, ACIAR Monograph (2008). p. 21–36.

[9] Mehmood K Zhang H Sabir AJ Abbas RZ Ijaz M Durrani AZ. A review on epidemiology, global prevalence and economical losses of fasciolosis in ruminants. Microb Pathog. (2017) 109:253–62. 10.1016/j.micpath.2017.06.006 28602837

[10] Mas-Coma S Bargues MD Valero MA. Diagnosis of human fascioliasis by stool and blood techniques: update for the present global scenario. Parasitology. (2014) 141:1918–46. 10.1017/S0031182014000869 25077569

[11] Mas-Coma S. Human fascioliasis emergence risks in developed countries: from individual patients and small epidemics to climate and global change impacts. Enf Emerg Microbio Clí*n*. (2020) 38:253–6. 10.1016/j.eimc.2020.01.014 32107024

[12] World Health Organization Sustaining the drive to overcome the global impact of neglected tropical diseases. Geneva: Department of Control of Neglected Tropical Diseases; World Health Organization; WHO Headquarters (2013). p. 128.

[13] Valero MA Bargues MD Khoubbane M Artigas P Quesada C Berinde L. Higher physiopathogenicity by *Fasciola gigantica* than by the genetically close *F. hepatica*: experimental long-term follow-up of biochemical markers. Trans Roy Soc Trop Med Hyg. (2016) 110:55–66. 10.1093/trstmh/trv110 26740363

[14] Malandrini JB Carnevale S Velazquez J Soria CC Diagnóstico de *Fasciola hepatica* con la técnica de ELISA en el Departamento de Tinogasta. Ciencia. (2009) 4:143–51.

[15] Mera y Sierra R Agramunt VH Cuervo P Mas-Coma S. Human fascioliasis in Argentina: retrospective overview, critical analysis and baseline for future research. Parasit Vector. (2011) 4:104. 10.1186/1756-3305-4-104 21663691 PMC3141741

[16] Bargues MD Malandrini JB Artigas P Soria CC Velasquez JN Carnevale S. Human fascioliasis endemic areas in Argentina: multigene characterisation of the lymnaeid vectors and climatic-environmental assessment of the transmission pattern. Parasit Vector. (2016) 9:306. 10.1186/s13071-016-1589-z 27229862 PMC4882814

[17] Hillyer GV Soler de Galanes M Rodriguez-Perez J Bjorland J Silva de Lagrava M Ramirez Guzman S. Use of the falcon assay screening test—enzyme-linked immunosorbent assay (FAST-ELISA) and the enzyme-linked immunoelectrotransfer blot (EITB) to determine the prevalence of human fascioliasis in the Bolivian Altiplano. Am J Trop Med Hyg. (1992) 46:603–9. 10.4269/ajtmh.1992.46.603 1599055

[18] Bjorland J Bryan RT Strauss W Hillyer GV McAuley JB. An outbreak of acute fascioliasis among Aymara Indians in the Bolivian Altiplano. Clin Inf Dis. (1995) 21:1228–33. 10.1093/clinids/21.5.1228 8589147

[19] Esteban JG Flores A Angles R Strauss W Aguirre C Mas-Coma S. A population-based coprological study of human fascioliasis in a hyperendemic area of the Bolivian Altiplano. Trop Med Int Health. (1997) 2:695–99. 10.1046/j.1365-3156.1997.d01-356.x 9270738

[20] Esteban JG Gonzalez C Bargues MD Angles R Sanchez C Naquira C. High fascioliasis infection in children linked to a man-made irrigation zone in Peru. Trop Med Int Health. (2002) 7:339–48. 10.1046/j.1365-3156.2002.00870.x 11952950

[21] Espinoza JR Maco V Marcos L Saez S Neyra V Terashima A. Evaluation of Fas2-ELISA for the serological detection of *Fasciola hepatica* infection in humans. Am J Trop Med Hyg. (2007) 76:977–82. 10.4269/ajtmh.2007.76.977 17488926

[22] Gonzalez LC Esteban JG Bargues MD Valero MA Ortiz P Naquira C. Hyperendemic human fascioliasis in Andean valleys: an altitudinal transect analysis in children of Cajamarca province, Peru. Acta Trop. (2011) 120:119–29. 10.1016/j.actatropica.2011.07.002 21767521

[23] Trueba G Guerrero T Fornasini M Casariego I Zapata S Ontaneda S. Detection of *Fasciola hepatica* infection in a community located in the Ecuadorian Andes. Am J Trop Med Hyg. (2000) 62:518. 10.4269/ajtmh.2000.62.518 11220770

[24] Bargues MD Gonzalez C Artigas P Mas-Coma S. A new baseline for fascioliasis in Venezuela: lymnaeid vectors ascertained by DNA sequencing and analysis of their relationships with human and animal infection. Parasit Vector. (2011) 4:200. 10.1186/1756-3305-4-200 21999170 PMC3213164

[25] Bargues MD Artigas P Khoubbane M Mas-Coma S. DNA sequence characterisation and phylogeography of *Lymnaea cousini* and related species, vectors of fascioliasis in northern Andean countries, with description of *L. meridensis* n. sp. (Gastropoda: Lymnaeidae). Parasit Vector. (2011) 4:132. 10.1186/1756-3305-4-132 21749718 PMC3168421

[26] Zumaquero-Rios JL Sarracent-Perez J Rojas-Garcia R Rojas-Rivero L Martinez-Tovilla Y Valero MA. Fascioliasis and intestinal parasitoses affecting schoolchildren in Atlixco, Puebla state, Mexico: epidemiology and treatment with nitazoxanide. PLoS Negl Trop Dis. (2013) 7:e2553. 10.1371/journal.pntd.0002553 24278492 PMC3836726

[27] Pritsch IC Molento MB Recount of reported cases of human fascioliasis in Brazil over the last 60 years. Rev Patol Trop. (2018) 47:75–85. 10.5216/rpt.v47i2.53636

[28] Mas-Coma S Angles R Esteban JG Bargues MD Buchon P Franken M. The northern Bolivian Altiplano: a region highly endemic for human fascioliasis. Trop Med Int Health. (1999) 4:45467. 10.1046/j.1365-3156.1999.00418.x 10444322

[29] Fuentes MV Valero MA Bargues MD Esteban JG Angles R Mas-Coma S. Analysis of climatic data and forecast indices for human fascioliasis at very high altitude. Ann Trop Med Parasitol. (1999) 93:835–50. 10.1080/00034983.1999.11813491 10715678

[30] Fuentes MV Malone JB Mas-Coma S. Validation of a mapping and predicting model for human fasciolosis transmission in Andean very high altitude endemic areas using remote sensing data. Acta Trop. (2001) 79:87–95. 10.1016/S0001-706X(01)00106-1 11378145

[31] Ollerenshaw CB The ecology of the liver fluke (*Fasciola hepatica*). Vet Rec. (1959) 71:957–65.

[32] Fox NJ White PCL McClean CJ Marion G Evans A Hutchings MR. Predicting impacts of climate change on *Fasciola hepatica* risk. PLoS ONE. (2011) 6:e16126. 10.1371/journal.pone.0016126 21249228 PMC3018428

[33] Afshan K Fortes-Lima CA Artigas P Valero MA Qayyum M Mas-Coma S. Impact of climate change and man-made irrigation systems on the transmission risk, long-term trend and seasonality of human and animal fascioliasis in Pakistan. Geospat Health. (2014) 8:317–34. 10.4081/gh.2014.22 24893010

[34] Silva AEP Freitas CC Dutra LV Molento MB. Assessing the risk of bovine fasciolosis using linear regression analysis for the state of Rio Grande do Sul, Brazil. Vet Parasitol. (2016) 217:7–13. 10.1016/j.vetpar.2015.12.021 26827853

[35] De NV Le TH Agramunt VH Mas-Coma S. Early postnatal and preschool age infection by *Fasciola* spp.: report of five cases from Vietnam and worldwide review. Am J Trop Med Hyg. (2020) 103:1578–89. 10.4269/ajtmh.20-0139 32618259 PMC7543854

[36] Villegas F Angles R Barrientos R Barrios G Valero MA Hamed K. Administration of triclabendazole is safe and effective in controlling fascioliasis in an endemic community of the Bolivian Altiplano. PLoS Negl Trop Dis. (2012) 6:e1720. 10.1371/journal.pntd.0001720 22880138 PMC3413701

[37] Valero MA Periago MV Perez-Crespo I Angles R Villegas F Aguirre C. Field evaluation of a coproantigen detection test for fascioliasis diagnosis and surveillance in human hyperendemic areas of Andean countries. PLoS Negl Trop Dis. (2012) 6:e1812. 10.1371/journal.pntd.0001812 23029575 PMC3441400

[38] Bargues MD Artigas P Angles R Osca D Duran P Buchon P. Genetic uniformity, geographical spread and anthropogenic habitat modifications of lymnaeid vectors found in a one health initiative in the highest human fascioliasis hyperendemic of the Bolivian Altiplano. Parasit Vector. (2020) 13:171. 10.1186/s13071-020-04045-x 32252808 PMC7137187

[39] Rinaldi L Gonzalez S Guerrero J Carol Aguilera L Musella V Genchi C. A one-health integrated approach to control fascioliasis in the Cajamarca valley of Peru. Geospat Health. (2012) 6:S67–73. 10.4081/gh.2012.124 23032285

[40] Webster JP Gower CM Knowles SCL Molyneux DH Fenton A. One health—an ecological and evolutionary framework for tackling neglected zoonotic diseases. Evol Appl. (2016) 9:313–33. 10.1111/eva.12341 26834828 PMC4721077

[41] Destoumieux-Garzón D Mavingui P Boetsch G Boissier J Darriet F Duboz P. The one health concept: 10 years old and a long road ahead. Front Vet Sci. (2018) 5:14. 10.3389/fvets.2018.00014 29484301 PMC5816263

[42] Boray JC Enigk K. Laboratory studies on the survival and infectivity of *Fasciola hepatica* and *F. gigantica* metacercariae. Z Tropenmed Parasitol. (1964) 15:324–31. 14316630

[43] Mas-Coma S Funatsu IR Bargues MD. *Fasciola hepatica* and lymnaeid snails occurring at very high altitude in South America. Parasitology. (2001) 123:S115–27. 10.1017/S0031182001008034 11769277

[44] Bargues MD Gayo V Sanchis J Artigas P Khoubbane M Birriel S. DNA multigene characterization of *Fasciola hepatica* and *Lymnaea neotropica* and its fascioliasis transmission capacity in Uruguay, with historical correlation, human report review and infection risk analysis. PLoS Negl Trop Dis. (2017) 11:e0005352. 10.1371/journal.pntd.0005352 28158188 PMC5310921

[45] Bargues MD Artigas P Khoubbane M Flores R Glöer P Rojas-Garcia R. *Lymnaea schirazensis*, an overlooked snail distorting fascioliasis data: genotype, phenotype, ecology, worldwide spread, susceptibility, applicability. PLoS ONE. (2011) 6:e24567. 10.1371/journal.pone.0024567 21980347 PMC3183092

[46] Bargues MD Artigas P Mera y Sierra R Pointier JP Mas-Coma S Characterisation of Lymnaea cubensis L. *viatrix* and *L. neotropica* n. sp., the main vectors of *Fasciola hepatica* in latin America, by analysis of their ribosomal and mitochondrial DNA. Ann Trop Med Parasitol. (2007) 101:621–41. 10.1179/136485907X229077 17877881

[47] Valero MA Panova M Comes AM Fons R Mas-Coma S. Patterns in size and shedding of *Fasciola hepatica* eggs by naturally and experimentally infected murid rodents. J Parasitol. (2002) 88:308–13. 10.1645/0022-3395(2002)088[0308:PISASO]2.0.CO;2 12054003

[48] Valero MA Mas-Coma S. Comparative infectivity of *Fasciola hepatica* metacercariae from isolates of the main and secondary reservoir animal host species in the Bolivian Altiplano high human endemic region. Folia Parasitol. (2000) 47:17–22. 10.14411/fp.2000.004 10833011

[49] Valero MA Santana M Morales M Hernandez JL Mas-Coma S. Risk of gallstone disease in advanced chronic phase of fascioliasis: an experimental study in a rat model. J Infect Dis. (2003) 188:787–93. 10.1086/377281 12934197

[50] Dennis WR Stone WM Swanson LE. A new laboratory and field diagnostic test for fluke ova in feces. J Am Vet Med Ass. (1954) 124:47–50. 13117747

[51] Jackson MR Mayhew TM Haas JD. The volumetric composition of human term placentae: altitudinal, ethnic and sex differences in Bolivia. J Anat. (1987) 152:173–87. 3654368 PMC1261755

[52] Frisancho D Frisancho O. Fisiología y patología digestiva en gran altitud. Rev Gastroenterol Peru. (1992) 121:55–158. 1340247

[53] Levine BD Kubo K Kobayashi T Fukushima M Shibamoto T Ueda G. Role of barometric pressure in pulmonary fluid balance and oxygen transport. J Appl Physiol. (1988) 64:419–28. 10.1152/jappl.1988.64.1.419 3356658

[54] Sarybaeva DV Zakharova LA Vasilenko AM Mikhailova AA Tulebekov BT. The immunomodulating action of myelopeptides in hypoxic hypoxia in animals. Biull Eksp Biol Med. (1988) 106:691–2. (in Russian). 10.1007/BF00802178 3207877

[55] Sakai A Ueda G Kobayashi T Kubo K Fukushima M Yoshimura K. Effects of elevated-hematocrit levels on pulmonary circulation in conscious sheep. Jap J Physiol. (1984) 34:871–82. 10.2170/jjphysiol.34.871 6241951

[56] Hirai K Kobayashi T Kubo K Shibamoto T. Effects of hypobaria on lung fluid balance in awake sheep. J Appl Physiol. (1988) 64:243–8. 10.1152/jappl.1988.64.1.243 3356641

[57] Dawes B Hughes DL. Fascioliasis: the invasive stages of *Fasciola hepatica* in mammalian hosts. Adv Parasitol. (1964) 2:97–168. 10.1016/S0065-308X(08)60587-4 14321784

[58] Boray JC Experimental fascioliasis in Australia. Adv Parasitol. (1969) 8:95–210. 10.1016/S0065-308X(08)60435-2 4935272

[59] Rowcliffe SA Ollerenshaw CB. Observations on the bionomics of the egg of *Fasciola hepatica*. Ann Trop Med Parasitol. (1960) 54:172–81. 10.1080/00034983.1960.11685973 14439674

[60] Boyunaga H Schmitz MGJ Brouwers JFHM Van Hellemond JJ Tielens AGM. *Fasciola hepatica* miracidia are dependent on respiration and endogenous glycogen degradation for their energy generation. Parasitology. (2001) 122:169–73. 10.1017/S0031182001007211 11272647

[61] Valero MA Marcos MD Comes AM Sendra M Mas-Coma S. Comparison of adult liver flukes from highland and lowland populations of Bolivian and Spanish sheep. J Helminthol. (1999) 73:341–45. 10.1017/S0022149X99000578 10654404

[62] Valero MA Panova M Mas-Coma S. Developmental differences in the uterus of *Fasciola hepatica* between livestock liver fluke populations from Bolivian highland and European lowlands. Parasitol Res. (2001) 87:337–42. 10.1007/PL00008588 11355685

[63] Valero MA Panova M Perez-Crespo I Khoubbane M Mas-Coma S. Correlation between egg-shedding and uterus development in *Fasciola hepatica* human and animal isolates: applied implications. Vet Parasitol. (2011) 183:79–86. 10.1016/j.vetpar.2011.07.003 21802206

[64] McGonigle S Dalton JP. Isolation of *Fasciola hepatica* haemoglobin. Parasitology. (1995) 111:209–15. 10.1017/S0031182000064969 7675536

[65] Ross IC McKay AC The bionomics of *Fasciola hepatica* in New South Wales and the intermediate host *Limnaea brazieri*. Bull Counc Sc Ind Res Aust. (1929) 5:1–63.

[66] Roberts WE Studies on the life-cycle of *Fasciola hepatica* (Linnaeus) and of its snail host *Limnaea* (Galba) *truncatula* (Müller) in the field and under controlled conditions in the laboratory. Ann Trop Med Parasitol. (1950) 44:187–206. 10.1080/00034983.1950.11685441 24538002

[67] Diez-Baños MA Rojo-Vázquez FA Influencia de la temperatura en el desarrollo de los huevos de *Fasciola hepatica*. An Fac Vet León. (1976) 22:65–75.

[68] Valenzuela G. Estudio epidemiológico acerca del desarrollo de huevos de *Fasciola hepatica* en el medio ambiente en Valdivia, Chile. Bol Chil Parasit. (1979) 34:31–5. 540082

[69] Wilson RA Smith G Thomas MR Fascioliasis. In: Anderson RM, editor. The Population Dynamics of Infectious Diseases: Theory and Applications. London; New York, NY: Chapman and Hall (1982). p. 262–319.

[70] Rondelaud D Variabilité interpopulationelle de l'infestation fasciolenne chez le molllesque *Lymnaea truncatula* Müller. Influence du contact préalable de la population avec le parasite. Bull Soc Zool France. (1993) 118:185–93.

[71] Vignoles P Dreyfuss G Rondelaud D. Larval development of *Fasciola hepatica* in experimental infections: variations with populations of *Lymnaea truncatula*. J Helminthol. (2002) 76:179–83. 10.1079/JOH2002112 12015832

[72] Kendall SB. Nutritional factors affecting the rate of development of *Fasciola hepatica* in *Limnaea truncatula*. J Helminthol. (1993) 23:179–90. 10.1017/S0022149X00032491 15409355

[73] Dreyfuss G Vignoles P Rondelaud D. *Fasciola hepatica*: the infectivity of cattle-origin miracidia had increased over the past years in central France. Parasitol Res. (2007) 101:115–60. 10.1007/s00436-007-0580-1 17522892

[74] Rondelaud D Barthe D Les générations rédiennes de *Fasciola hepatica* L. Premières observations chez des Limnées tronquées en fin de cycle parasitaire. Bull Soc Franç Parasitol. (1986) 4:29–38.

[75] Rondelaud D Dreyfuss G *Fasciola hepatica*: the influence of the definitive host on the characteristics of the infection in the snail *Lymnaea truncatula*. Parasite. (1995) 2:275–80. 10.1051/parasite/1995023275

[76] Dreyfuss G Rondelaud D. *Fasciola hepatica*: a study of the shedding of cercariae from *Lymnaea truncatula* raised under constant conditions of temperature and photoperiod. Parasite. (1994) 1:401–4. 10.1051/parasite/1994014401 9140507

[77] Hodasi JKM. The output of cercariae of *Fasciola hepatica* by *Lymnaea truncatula* and the distribution of metacercariae on grass. Parasitology. (1972) 63:431–56. 5010460 10.1017/s0031182000044644

[78] Audousset JC Rondelaud D Dreyfuss G Vareille-Morel C Les émissions cercariennes de *Fasciola hepatica* L. chez le mollusque *Lymnaea truncatula* Müller. A propos de quelques observations chronobiologiques. Bull Soc Fr Parasitol. (1989) 7:217–24.

[79] Bargues MD Oviedo JA Funatsu IR Rodriguez A Mas-Coma S Survival of lymnaeid snails from the Bolivian Northern Altiplano after the parasitation by different Bolivian isolates of *Fasciola hepatica* (Linnaeus, 1758) (Trematoda: Fasciolidae). In: Guerra A, Rolán E, Rocha F, editors. Unitas Malacologica. Vigo: Instituto de Investigaciones Marinas; CSIC (1995). p. 443–5.

[80] Kimura S Shimizu A Studies on the survival and infectivity of *Fasciola gigantica* metacercariae. Sci Rept Fac Agr Kobe Univ. (1979) 13:347–49.

[81] Dixon KE. The relative suitability of sheep and cattle as hosts for the liver fluke, *Fasciola hepatica* L. J Helminthol. (1964) 38:203–12. 10.1017/S0022149X00033782 14250808

[82] Knight RA. Experimental cross infections of *Fasciola hepatica* in lambs and calves. J Parasitol. (1978) 64:601–5. 10.2307/3279941 28389

[83] Valero MA Darce NA Panova M Mas-Coma S. Relationships between host species and morphometric patterns in *Fasciola hepatica* adults and eggs from the northern Bolivian Altiplano hyperendemic region. Vet Parasitol. (2001) 102:85–100. 10.1016/S0304-4017(01)00499-X 11705655

[84] Hervé D Rojas A Vías de intensificación de la ganadería bovina en el Altiplano Boliviano. La Paz: Instituto Francés de Investigacion Cientifica para el Desarrollo en Cooperación (ORSTOM) y Cooperación Técnica de la Iglesia Danesa (DANCHURCHAID); Artes Gráficas Potosí (1994). p. 205.

[85] Mas-Coma S Angles R Strauss W Esteban JG Oviedo JA Buchon P Human fasciolasis in Bolivia: a general analysis and a critical review of existing data. Res Rev Parasitol. (1995) 55:73–93.

[86] Mas-Coma S Valero MA Bargues MD. *Fasciola*, lymnaeids and human fascioliasis, with a global overview on disease transmission, epidemiology, evolutionary genetics, molecular epidemiology and control. Adv Parasitol. (2009) 69:41–146. 10.1016/S0065-308X(09)69002-3 19622408

[87] Twomey AJ Berry DP Evans RD Doherty ML Graham DA Purfield DC. Genome-wide association study of endo-parasite phenotypes using imputed whole-genome sequence data in dairy and beef cattle. Genet Sel Evol. (2019) 51:15. 10.1186/s12711-019-0457-7 30999842 PMC6471778

[88] Ueno H Arandia R Morales G Medina G. Fascioliasis of livestock and snail host for *Fasciola* in the Altipiano region of Bolivia. Nat Inst Anim Health Quart. (1975) 15:61–7. 1182037

[89] Hillyer GV Soler de Galanes M Buchon P Bjorland J. Herd evaluation by enzyme-linked irnmunosorbent assay for the determination of *Fasciola hepatica* infection in sheep and cattle from the Altiplano of Bolivia. Vet Parasitol. (1996) 61:211–20. 10.1016/0304-4017(95)00831-4 8720559

[90] Valero MA Perez-Crespo I Chillon-Marinas C Khoubbane M Quesada C Reguera-Gomez M. *Fasciola hepatica* reinfection potentiates a mixed Th1/Th2/Th17/Treg response and correlates with the clinical phenotypes of anemia. PLoS ONE. (2017) 12:e0173456. 10.1371/journal.pone.0173456 28362822 PMC5376296

[91] Valero MA Girones N Reguera-Gomez M Perez-Crespo I Lopez-Garcia MP Quesada C. Impact of fascioliasis reinfection on *Fasciola hepatica* egg shedding: relationship with the immune-regulatory response. Acta Trop. (2020) 209:105518. 10.1016/j.actatropica.2020.105518 32371223

[92] Ubeira FM Muiño L Valero MA Periago MV Perez-Crespo I Mezo M. MM3-ELISA detection of *Fasciola hepatica* coproantigens in preserved human stool samples. Am J Trop Med Hyg. (2009) 81:156–62. 10.4269/ajtmh.2009.81.156 19556582

[93] Valero MA Perez-Crespo I Khoubbane M Artigas P Panova M Ortiz P *Fasciola hepatica* phenotypic characterisation in Andean human endemic areas: valley versus altiplanic patterns analysed in liver flukes from sheep from Cajamarca and Mantaro, Peru. Infect Genet Evol. (2012) 12:403–10. 10.1016/j.meegid.2012.01.009 22285769

[94] Valero MA Ubeira FM Khoubbane M Artigas P Muiño L Mezo M. MM3-ELISA evaluation of coproantigen release and serum antibody production in sheep experimentally infected with *Fasciola hepatica* and *F*. gigantica. Vet Parasitol. (2009) 159:77–81. 10.1016/j.vetpar.2008.10.014 19019548

